# Major Intraoperative Complications During Minimally Invasive Esophagectomy

**DOI:** 10.1245/s10434-023-14340-3

**Published:** 2023-10-02

**Authors:** H. Söderström, J. Moons, P. Nafteux, E. Uzun, P. Grimminger, M. D. P. Luyer, G. A. P. Nieuwenhuijzen, M. Nilsson, M. Hayami, S. Degisors, G. Piessen, H. Vanommeslaeghe, E. Van Daele, E. Cheong, Ch A. Gutschow, D. Vetter, N. Schuring, S. S. Gisbertz, J. Räsänen

**Affiliations:** 1 Department of Thoracic Surgery, Helsinki University Hospital, University of Helsinki, Helsinki, Finland; 2 Department of Thoracic Surgery, University Hospitals Leuven, Leuven, Belgium; 3 Laboratory of Respiratory Diseases and Thoracic Surgery (BREATHE), Department of Chronic Diseases, Metabolism and Ageing, KU Leuven, Leuven, Belgium; 4 Department of General, Visceral and Transplant Surgery, University Medical Center of the Johannes Gutenberg University, Mainz, Germany; 5 Department of Surgery, Catharina Hospital, Eindhoven, The Netherlands; 6 Department of Upper Abdominal Surgery, Center for Digestive Diseases, Karolinska University Hospital, Stockholm, Sweden; 7 Division of Surgery, Department of Clinical Science, Intervention and Technology (CLINTEC), Karolinska Institutet, Stockholm, Sweden; 8 Department of Gastroenterological Surgery, Gastroenterological Center, Cancer Institute Hospital, Japanese Foundation for Cancer Research, Tokyo, Japan; 9 Department of Digestive and Oncological Surgery, University Hospital C. Huriez Place de Verdun, Lille Cedex, France; 10 Department of Gastro-Intestinal Surgery, Ghent University Hospital, Ghent, Belgium; 11 Norfolk and Norwich University Hospital NHS FT, Norwich, UK; 12 Department of Surgery and Transplantation, University Hospital Zurich, Zurich, Switzerland; 13 Department of Surgery, Amsterdam UMC Location University of Amsterdam, Amsterdam, The Netherlands; 14 Cancer Treatment and Quality of Life, Cancer Center Amsterdam, Amsterdam, The Netherlands

**Keywords:** Complication, Surgical outcome, Esophageal cancer, Minimally invasive esophagectomy

## Abstract

**Background:**

Studies have shown minimally invasive esophagectomy (MIE) to be a feasible surgical technique in treating esophageal carcinoma. Postoperative complications have been extensively reviewed, but literature focusing on intraoperative complications is limited. The main objective of this study was to report major intraoperative complications and 90-day mortality during MIE for cancer.

**Methods:**

Data were collected retrospectively from 10 European esophageal surgery centers. All intention-to-treat, minimally invasive laparoscopic/thoracoscopic esophagectomies with gastric conduit reconstruction for esophageal and GE junction cancers operated on between 2003 and 2019 were reviewed. Major intraoperative complications were defined as loss of conduit, erroneous transection of vascular structures, significant injury to other organs including bowel, heart, liver or lung, splenectomy, or other major complications including intubation injuries, arrhythmia, pulmonary embolism, and myocardial infarction.

**Results:**

Amongst 2862 MIE cases we identified 98 patients with 101 intraoperative complications. Vascular injuries were the most prevalent, 41 during laparoscopy and 19 during thoracoscopy, with injuries to 18 different vessels. There were 24 splenic vascular or capsular injuries, 11 requiring splenectomies. Four losses of conduit due to gastroepiploic artery injury and six bowel injuries were reported. Eight tracheobronchial lesions needed repair, and 11 patients had significant lung parenchyma injuries. There were 2 on-table deaths. Ninety-day mortality was 9.2%.

**Conclusions:**

This study offers an overview of the range of different intraoperative complications during minimally invasive esophagectomy. Mortality, especially from intrathoracic vascular injuries, appears significant.

Several studies have shown minimally invasive esophagectomy (MIE) to be a feasible surgical technique in treating esophageal carcinoma.^1–3^ MIE has been shown to have comparable, even superior, immediate postoperative outcomes without compromising long-term oncological results compared with open esophagectomy (OE).^2–4^ There is an increasing implementation of MIE in Europe, and the rate increased from 20% in 2007 to almost 50% in 2014.^5^ It is the standard approach in some countries.^6^

Postoperative complications for MIE have been recently reviewed in a meta-analysis^7^ and by a multicenter study that benchmarked outcomes.^8^ Studies show MIE to be a challenging procedure with relatively high postoperative morbidity.^7–9^ The advantages of MIE over OE include fewer overall, but especially pulmonary, complications, shortened length of stay and improved short-term quality of life,^2,3^ but the procedure is associated with a significant learning curve.^9,10^ The available literature focuses exclusively on postoperative outcomes, and while several studies on MIE^11–14^ mention intraoperative complications (IOCs), the literature on these complications is limited. We can assume intraoperative adverse events can lead to postoperative complications, so it would be imperative to reduce IOCs to improve overall surgical outcomes.

Hence, the main objective of this retrospective multinational study was to report major intraoperative complications and associated unplanned surgery, types of injury repair, and 90-day mortality during MIE for cancer.

## Methods

An invitation to join the study was sent to 19 esophageal surgery centers in 9 European countries. Participating centers were required to have > 3 years’ experience with minimally invasive (thoracoscopic + laparoscopic) esophagectomy and have a prospective or otherwise thorough patient registry. Each center identified complication cases in their institutional patient registries for submission. The data were collected using an Excel table and pseudonymized before submission to the primary researchers. All consecutive patients who experienced IOCs during intention-to-treat MIE for esophageal and GE junction cancer were included in the study. The study period was from the initiation of each participating center MIE program until 31 October 2019.

Major intraoperative complications were defined as (1) loss of planned conduit; (2) sudden blood loss of > 500 ml; (3) erroneous transection of vascular structures; (4) significant injury to other organs including bowel, heart, liver, or lung; (5) splenectomy; (6) major anesthesia related injuries or complications; and (7) immediate life-threatening situations including arrhythmia, myocardial infarction, and pulmonary embolism. We also included minor intraoperative events that led to additional resections or repair within 5 days postoperatively. To ensure no significant complications were overlooked in the initial patient registry screening, all centers especially surveyed the cases with conversion, total blood loss over 500 ml, operative time more than 400 min and re-operation within 5 days postoperatively. Cases with no resection due to oncological reasons were excluded.

Gathered data included patient characteristics, neoadjuvant therapy, histology, clinical and pathological TNM, tumor site, date of surgery, surgical technique, major intraoperative complications as defined above, intraoperative blood transfusion and blood loss, type of injury repair, reason for complication if apparent, reason for conversion when applicable, additional unplanned surgery, length of stay, and 90-day mortality.

The complications were classified as either “laparoscopic” or “thoracoscopic” depending on their occurrence during the abdominal or the thoracic phase of the procedure, respectively. These categories were further divided into “visceral” or “vascular” depending on the injured structure or organ. Complications fitting none of the subcategories, including anesthesia related incidences, were classified as miscellaneous.

The study was approved by the Helsinki University Ethical board. Researchers at each center applied for required national research and ethical permits.

### Statistical Analyses

Characteristics of patients are reported using frequency and percentage of categorial variables. Incidences of specific complications are presented as frequency and percentage of each subgroup based on location and type of complication. Blood loss is reported as mean with range, and length of stay as median with interquartile range (IQR). Data were analyzed by using SPSS Statistics for Macintosh, Version 28.0 (IBM SPSS Statistics for Macintosh, Version 28.0. Armonk, NY: IBM Corp).

## Results

Data from 2862 patients who underwent MIE between 2003 and 2019 at 10 centers in eight European countries were reviewed and screened. The response rate from invited centers was 52.6%. A total of 98 (3.4%) patients with 101 IOC events were identified and submitted for analysis. A conversion rate of 49% (*N* = 48) was noted, and 36.7% (*N* = 36) of the patients were converted due to a complication. There were two intraoperative deaths. Patient demographics and tumor characteristics are presented in Table 1. Hospital length of stay and mortality are shown in Table 2.

**Table 1:** Patient and tumor characteristics (*N* = 98)

Age (years)^a^	66 (8.2)
Gender (*N*; %)	
Male	77 (78.6 %)
Female	21 (21.4 %)
ASA classification (*N*; %)	
1–2	64 (65.3 %)
3	33 (33.7 %)
ECOG status (*N*; %)	
0	74 (75.5 %)
1–2	24 (24.5 %)
BMI^a^	24.7 (3.8)
Neoadjuvant treatment (*N*; %)	65 (66.3 %)
Chemotherapy	16 (16.3 %)
Chemoradiotherapy	49 (50.0 %)
Tumor histology (*N*; %)	
Adenocarcinoma	79 (80.6 %)
Squamous cell carcinoma	19 (19.4 %)
Clinical stage (*N*; %)^b^	
I	19 (19.4 %)
IIA-B	16 (16.3 %)
III	44 (45.9 %)
IVA-B	17 (17.4 %)
Not specified	1 (1.0 %)
pN positive (*N*; %)	35 (35.7 %)
Surgical technique (*N*; %)	
Ivor Lewis	59 (60.2 %)
McKeown	39 (39.8 %)
Prone position (*N*; %)	46 (46.9 %)

^a^Mean (SD)

^b^According to AJCC 8th edition Classification

*BMI*, body mass index (kg/m^2^); *ASA*, American Society of Anesthesiologists physical status *classification; ECOG*, Eastern Cooperative Oncology Group Performance Status

**Table 2:** Re-operation rates, length of stay (LOS) and mortality

Re-operation within 5 days	9.2 % (*N* = 9)
ICU LOS (days)^a^	2 $(6)$
Hospital LOS (days)^a^	14.5 $(17.3)$
90-day mortality (*N* = 98)	9.2 % (9/98)
Injury specific 90-day mortality (*N* = 101)^b^	
Laparoscopic–vascular (*N* = 41)	7.3 % (3/41)
Laparoscopic–visceral (*N* = 12)	0 %
Thoracoscopic–vascular (*N* = 19)	21.1 % (4/19)
Thoracoscopic–visceral (*N* = 18)	11.1 % (2/18)
Miscellaneous (*N* = 11)	9 % (1/11)

^a^Median; IQR, interquartile range

^b^%, number of deaths/patients in subcategory

### Complications During Laparoscopic Phase

**Fig. 1:**
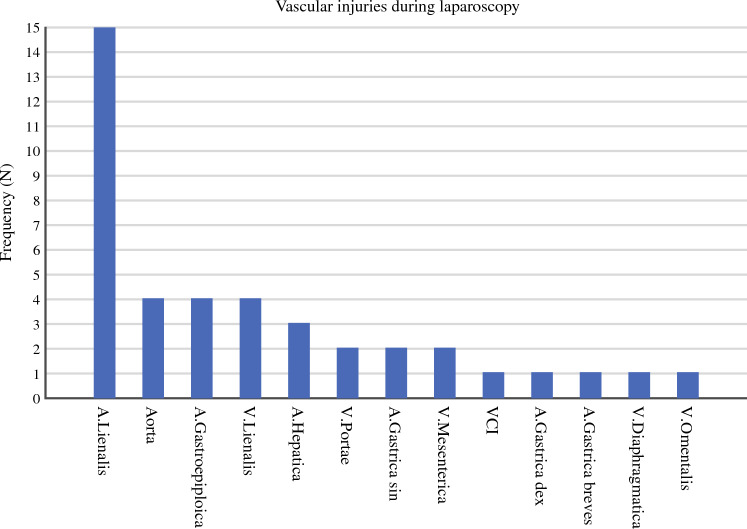
Vascular injuries during laparoscopy

During the laparoscopic phase, some 41 vascular complications were identified with injuries to 13 different vessels described (Fig. 1). The most frequently injured vessel was the splenic artery (*N* = 15) followed by the splenic vein (*N* = 4), gastroepiploic artery (*N* = 4) and aorta (*N* = 4). Four (9.7%) patients underwent a change of the planned conduit due to a gastroepiploic artery injury, including two coloplasties, one jejunal reconstruction, and one intrathoracic short tubulate gastric conduit. Amongst all abdominal vascular injuries, 12 (29.2%) were repaired by suturing, nine (21.9%) with clips, three (7.3%) with hemostats, one (2.4%) with cautery and for one (2.4%) a.gastrica breves injury type of repair was not described. Amongst the 15 splenic artery injuries, eight required a splenectomy and three were repaired with a vascular anastomosis. Mean estimated blood loss was 1240 ml (range 50–8100 ml). Conversion to laparotomy was required in 43.9% (*N* = 18) of these patients and in 34.1% (*N* = 14) the complication was deemed immediately life-threatening. For this sub-group of patients, 90-day mortality was 7.3% (*N* = 3) (Table 2).

**Fig. 2:**
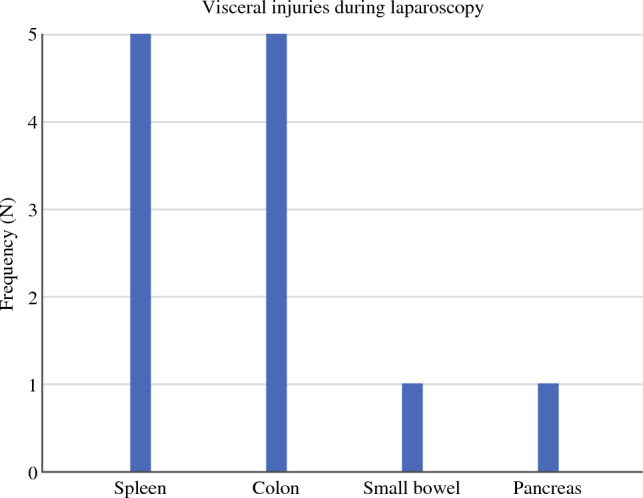
Visceral injuries during laparoscopy

Visceral injuries during laparoscopy were the least prevalent in the cohort (*N* = 12). These included five spleen injuries, of which three required splenectomy; five colon injuries, of which three were sutured and two were resected; one small-bowel lesion requiring resection, and one pancreatic injury requiring sutures. The types of injuries are shown in Fig. 2. Mean estimated blood loss was 380 ml (range 50–1500 ml). In this category, 33.4% (*N* = 4) of the cases were converted to laparotomy, 16.7% (*N* = 2) required re-operation within 24 h and in 8.3% (*N* = 1) of the cases the IOC was deemed immediately life-threatening. There was no 90-day mortality in this group (Table 2).

### Complications During Thoracoscopic Phase

**Fig. 3:**
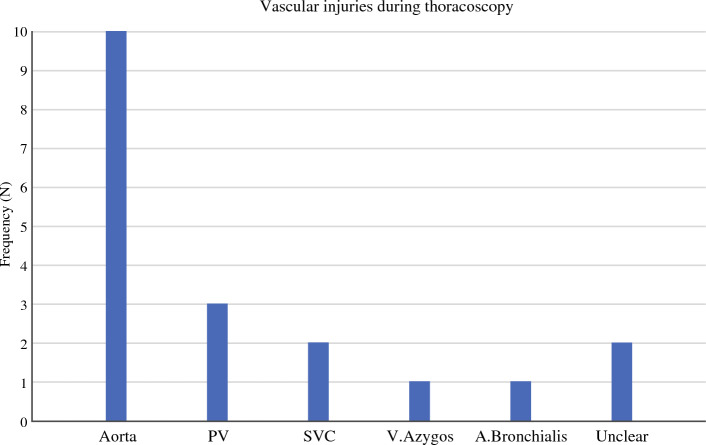
Vascular injuries during thoracoscopy

During the thoracoscopic phase a total of 19 vascular complications were identified with injuries described to 5 different vessels (Fig. 3). The majority of the reported injuries were to the aorta (*N* = 10), followed by pulmonary vein (*N* = 3), superior vena cava (SVC) (*N* = 2), bronchial artery (*N* = 1), and azygos vein (*N* = 1). Amongst all vascular injuries in the chest, 12 (63.2%) were repaired by suturing, four (21.1%) by clipping, and two (10.5%) by hemostats and packing. One SVC injury required cardiopulmonary bypass for repair by suturing. Mean estimated blood loss was 1410 ml (range 200–4600 ml). Amongst these patients 52.6% (*N* = 10) required conversion to thoracotomy and 26.3% (*N* = 5) required re-operation within 24 h. The IOC was deemed immediately life-threatening in 78.9% (*N* = 15) of the patients. In this group, 90-day mortality was 21.1% (*N* = 4) including one on-table death from an aortic arch injury (Table 2). One patient died in hospital 150 days after primary surgery due to subsequent complications.

**Fig. 4:**
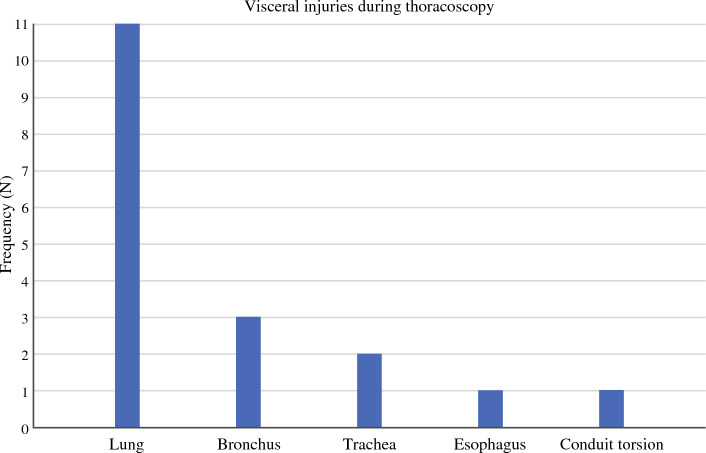
Visceral injuries during thoracoscopy

Some 18 visceral IOCs were identified during the thoracoscopic phase (Fig. 4). Of these, 11 were to lung parenchyma repaired either by suturing (*N* = 7), pleural drainage (*N* = 3), or wedge resection (*N* = 1). There were five injuries to the tracheobronchial tree; three were repaired by suturing and two required a muscle flap. In one case the stapling device injured the esophageal stump and had to be replaced. There was one case of conduit torsion that was discovered postoperatively, the patient required de-torsion and re-anastomosis before tolerating oral feeding. Mean estimated blood loss was 490 ml (range 100–1700 ml). A conversion to thoracotomy was required in 44.4% (*N* = 8) of the cases. None of the complications were deemed immediately life-threatening. Ninety-day mortality was 11.1% (*N* = 2) including one on-table death due to myocardial infarction. Both deaths were deemed unrelated to the reported IOC (Table 2).

### Anesthesia Related and Miscellaneous Complications

**Fig. 5:**
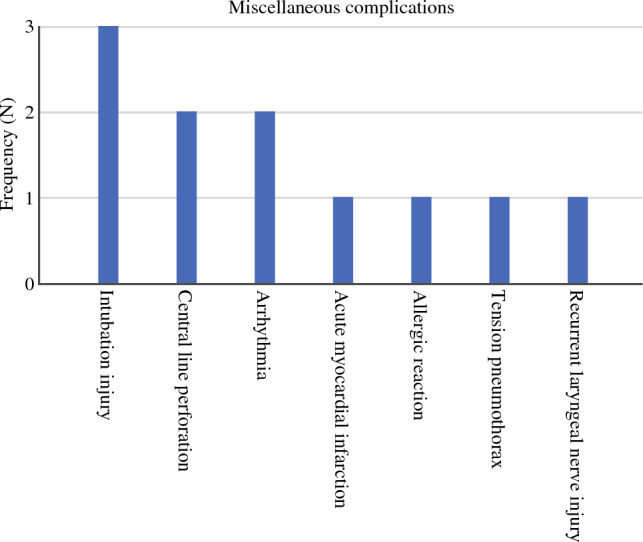
Miscellaneous complications

Complications that fit none of the abovementioned categories were labeled as “miscellaneous” (*N* = 11) (Fig. 5). There were three intubation injuries to the airways requiring either stenting or muscle flap repair and two conversions for central line perforations. One bilateral recurrent laryngeal nerve injury that required immediate re-intubation and ICU admission for dyspnea was noted, and one tension pneumothorax during the laparoscopic phase was treated with a chest tube insertion. One patient was admitted to the burn unit for allergic toxic necrolysis caused by prophylactic intraoperative antibiotic administration. There were two cases of malignant arrhythmias requiring resuscitation and cardioversion. One patient died on-table from an acute myocardial infarction despite conversion and open-heart massage. Mean estimated blood loss was 300 ml (range 100–500 ml). A conversion to thoracotomy was required in 54.5% (*N* = 6) of the cases. Three (27.3%) of the complications were deemed immediately life-threatening. Ninety-day mortality was 9.1% (*N* = 1) (Table 2).

Re-operation rates, lengths of stay, and mortalities are reported in Table 2.

## Discussion

This multi-center study reports the intraoperative complications encountered during MIE amongst 2862 esophageal cancer patients and offers a comprehensive overview of the wide range of different types of possible injuries. In our series, injuries during laparoscopy were slightly more common than during thoracoscopy. Overall, vascular lesions were more common than visceral injuries, they were associated with more blood loss, and were more frequently deemed as immediately life-threatening. Counting parenchymal injuries to the spleen, 67 out of the 102 reported injuries were related to bleeding. We show that all blood vessels in the surgical field are at risk for significant injury, with 18 different vascular lesions reported. There was significant 90-day mortality, especially amongst those suffering vascular injuries in the chest, compared with previously benchmarked results.^8^ Although the retrospective nature of this study does not provide exact mortality figures, we can say with confidence that on-table deaths are exceedingly rare (0.07%).

IOCs in minimally invasive thoracic surgery have been scarcely investigated. A European multicenter study showed a low 1.5% IOC rate in VATS lung resections that was not related to surgeon experience but had an important impact on patient outcome.^15^

Several studies mention intraoperative MIE complications, but do not define complications, nor specify injuries. The incidence, however, seems to be low.^11–14^ The only randomized trial comparing MIE and OE reports no conversions due to intraoperative complications and does not mention any IOCs in either group.^3^ Luketich et al. published a large series of 1011 MIE in 2012 noting no intraoperative mortality but some unanticipated intraoperative events including bleeding (1%), myocardial infarction (1%), and splenectomy (0.2%).^11^ A Dutch group reviewed 2598 patients (48% MIE) and reported 23 splenectomies (0.9%), 10 intestinal damages (0.4%), and seven (0.3%) damages to the trachea. The postoperative re-intervention rate was 23.2% including radiological, endoscopic, and surgical procedures. As this was a study on BMI/outcome association, no differentiation in outcome between surgical approaches was mentioned.^12^ In a recent study, Grimminger et al. compared semi-prone with left lateral thoracoscopy positioning in 141 consecutive patients and noted 6.38% and 5.32% IOC rates for the approaches, respectively. The group did not specify the complications, but there were no conversions for complications and no major vascular bleeding, airway injury, or anastomosis insufficiencies reported.^13^ The Brigham Esophageal Study Team reported surgical outcomes for 123 3-hole MIE patients and only noted one (0.8%) IOC in the entire cohort, a small-bowel injury requiring resection. There were, however, a significant number of blood transfusions reported during hospitalization (range 0–49 units), and bleeding was noted as a postoperative complication in four (3.3%) patients, including one reoperation for hemothorax.^14^ The complications described in the abovementioned studies were all found in our patient cohort. However, we found 65.7% of all reported complications to be related to bleeding. Only one of these studies reports bleeding as an unanticipated event,^11^ while the largest series notes no bleeding but a 0.9% splenectomy rate.^12^ This discrepancy could be explained by the lack of definition for significant intraoperative vessel injury. We included all injuries with more than 500 ml sudden blood loss, and those requiring intervention. This definition, in our opinion, accounts for situations that are potentially life-threatening if not promptly amended.

A large proportion of esophagectomies are still performed at low-volume centers which has been linked to a poorer outcome in several studies.^16–18^ In addition, esophagectomies are performed by a wide array of surgical specialties including thoracic surgeons, cardiothoracic surgeons, visceral/upper GI surgeons, surgical oncologists, and general surgeons, all with differing training backgrounds.^19^ Since intraoperative adverse events appear rare^11–14^ it can be assumed that many surgeons lack familiarity with the range of possible complications. As this study reports the different kinds of complications that may be encountered, it can help draw attention to the most hazardous parts of the operation and therefore increase surgical safety.

There are several limitations to this study, the main being its retrospective nature. Even though screening criteria were applied to ensure identification of complications, we still had to rely on operative notes and cannot assume all cases were recorded. Incidence can therefore not be concluded from this dataset. Another obvious limitation is the lack of a control group as we were unable to include the whole patient cohort in the analysis, limiting our ability to comment on IOC association with patient characteristics, postoperative morbidity, and cohort-specific mortality. A prospective study would provide more reliable data on incidence and correlation with outcomes. The strength of this study is the large cohort of patients that were not limited by type of neoadjuvant therapy or surgical approach. We identified a wide range of different types of complications, and this was not limited by the retrospective nature of the study. We are confident we included all on-table deaths.

In conclusion, this study sheds new light on different intraoperative complications and hazardous situations encountered during minimally invasive esophagectomy. Even though overall incidence appears low, mortality, especially from intrathoracic vascular injuries, can be quite significant.
